# Evolution characteristics and influencing factors of information network in Guangdong-Hong Kong-Macao Greater Bay Area

**DOI:** 10.1371/journal.pone.0298410

**Published:** 2024-05-17

**Authors:** Zhichen Yang, Yuxi Wu, Zilong Ma, Fangfang Wang, Rongjian Chen, Yixuan Wang, Zaoli Tian, Jiali Kuang, Yisen Chen, Aichun Chen

**Affiliations:** 1 School of Economics, Jinan University, Guangzhou, China; 2 School of Economics and Management, Beijing University of Technology, Beijing, China; 3 School of Economics, Guangdong University of Finance and Economics, Guangzhou, China; 4 School of Digital Economics, Guangdong University of Finance & Economics, Foshan, China; 5 School of International Economics and Trade, Guangdong Baiyun University, Guangzhou, China; 6 School of Cultural Tourism and Geography, Guangdong University of Finance and Economics, Guangzhou, China; Qufu Normal University, CHINA

## Abstract

In the context of the digital information era, the impact of "The Internet Plus," "Big Data," and other technologies on urban social development has been far beyond any preceding era, under the influence of information technology, urban agglomeration space exhibits a new layout. Based on the search engine data of eleven cities in the Guangdong-Hong Kong-Macao Greater Bay Area from 2012 to 2021, this research constructs the inter-city information network strength linkage matrix to examine the evolution characteristics of city network structure and its driving causes. The results reveal that (1) the overall information linkage strength exhibits a pattern of steadily growing the radiating effect from the leading cities of Guangdong, Shenzhen, and Hong Kong to the surrounding cities, and a closer and more balanced information linkage network is gradually built. (2) Guangzhou-Shenzhen-Hong Kong-Guangdong-Hong Kong-Macao Greater Bay Area information linkage absolute control advantage, four cities Foshan, Dongguan, Zhuhai, Macao regional hub position steadily highlighted. The entire information connection network of the urban agglomerations tends to be flat and polycentric at the same time. (3) The regional core-edge hierarchy is well established, with the four cities of Guangzhou, Dongguan, Shenzhen, and Hong Kong creating a northwest-southeast orientation. The core metropolis regions of Guangdong, Hong Kong, and Macao in the Greater Bay Area increasingly exert a radiation spreading effect to the northeast and southwest. (4) The urban economy, transportation distance, and information infrastructure have substantial effects on the information connection intensity network of urban clusters.

## Introduction

With the backing of modern information technology, "the Internet Plus" and "big data" have far-reaching consequences on urban society, economy, and culture. The impact of information technology on urban social development is significantly higher than ever before. Cities and urban agglomerations have traditionally played a significant role in the economic and social development of an area. Traditional urban geography, which studies changes in the spatial structure of actual urban sites in terms of physical entities, cannot adequately describe the complexities of the urban spatial structure under the various influencing variables such as "information flow" and "traffic flow" today. The complex alterations of the urban spatial structure are under the impact of numerous variables, such as "information flow" and "traffic flow." In the context of the digital age, the "location space’ of the international economy is being replaced by the "space of flows." The contemporary world economic system is a new spatial logic based on "flows’, nodes, and "networks"[1–3]. The leading role of information flows in the changing pattern of inter-city connections, the economic expansion of metropolitan areas, and the formation of new urban networks is becoming more clear.

The report of the 19th National Congress points out that the construction of the Guangdong-Hong Kong-Macao Greater Bay Area, cooperation among Guangdong, Hong Kong, and Macao, and Pan-Pearl River Delta (PPRD) regional cooperation will be the focus, comprehensively promote mutually beneficial cooperation between the Mainland and Hong Kong and Macao, actively promote the construction of the Bay Area, and advocate in-depth cooperation in all aspects of the PPRD region. In February 2019, the Outline of the Development Plan of the Guangdong-Hong Kong-Macao Greater Bay Area was officially released, based on the construction of a dynamic and internationally competitive first-class Bay Area and a world-class city cluster and building a model of high-quality development, Improving information infrastructure, strengthening smart city construction, creating world-class smart city clusters, promoting cross-domain synergistic smart city development, improving the coordination mechanism for city cluster development, and developing a model for high-quality development, elevating the construction of the Guangdong-Hong Kong-Macao Greater Bay Area to a new strategic height [4]. The "Three-Year Implementation Plan for Promoting New Infrastructure Construction in Guangdong Province (2020–2022)" was formally issued in November 2020, and the policy document stated that we should seize the opportunity to promote the construction of the Guangdong-Hong Kong-Macao Greater Bay Area as a world-class urban agglomeration, and by 2022, we should preliminarily form a new infrastructure system that is led by the new development concept, driven by scientific and technological innovation, and based on the information network, to support the digital transformation, intelligent upgrading, and convergence and innovation. The flow of information elements between regions has contributed to the emergence of a new pattern of regional development alongside the rise of digital information. Unlike physical ties, this virtual link between urban spatial associations and their evolution process is frequently overlooked in studies, and it is incapable of explaining the underlying mechanism of regional inter-city interactions under the new pattern. Therefore, the study of information networks in the Guangdong-Hong Kong-Macao Greater Bay Area is carried out to focus on inter-city differences from the integration of virtual society and reality level, and to propose theoretical suggestions for the direction of cooperation among Guangdong-Hong Kong-Macao cities, which response to the common needs of national city cluster construction and coordinated regional economic development in the technological era.

Based on the engine search volume data of 11 cities in the Guangdong-Hong Kong-Macao Greater Bay Area from 2012–2021, this article constructs an information linkage matrix to analyze the information linkage strength network in the Guangdong-Hong Kong-Macao Greater Bay Area. With reference to Yang et al. (2024), using centrality analysis, core-edge analysis, and QAP analysis [5], exploring the spatial structural characteristics and evolution laws of information networks, analyzing the impact of several influencing factors such as employment population, urban economic development, urban distance, information infrastructure construction, and scientific and technological education capacity on the strength of information links between cities, and providing useful guidance to support the construction of the Guangdong-Hong Kong-Macao Greater Bay Area Big Data Center, build a digital Greater Bay Area with a smooth flow of data elements, and promote coordinated regional governance.

## Literature review

Castells (1989) was the first to suggest a "new spatial logic," arguing that in gauging the relative importance of cities, the "mobile" character of contemporary inter-city linkages should be taken into account and that mobile space will replace the space of place [6]. As "mobility space" gradually becomes a new perspective for studying the spatial structure of urban agglomerations, many scholars have sought to move away from the hierarchical relationships of subordination, domination, and control in the previous literature to a network relationship of equality, sharing, cooperation, and complementarity [7–9]. Network theory in the discipline of sociology grew rapidly at this time and had a revolutionary impact in various fields, such as urban economics and spatial economics. The research direction of regional networks concentrated early on the structure and hierarchy of networks in related domains such as trade and commerce economies, air traffic, multinational enterprises, and international relations [10–13]. With the increasing innovation of information technology, information flow, represented by big data on the Internet, has steadily become a new hot spot in the study of regional integration [14]. Bao Z (2022) utilizes artificial intelligence technology to deeply mine and analyze the information flow to improve the efficiency and value of information flow utilization [15]. Chen et al.(2021) and Yu et al.(2023) explored the dynamic spatial evolution of the stock module using stock data [16, 17]. Wu and Huang (2023) use a time-zone vector autoregressive (VAR) model to study the co-movement of global financial markets, revealing the spatial transmission mechanism of information across continents during normal and turbulent times [18]. Yu and Huang (2023) Cross-sectional Uncertainty (CSU) has a significant ability to predict stock returns with a higher annual forecasting effect than the popular factors [19]. Chen et al. (2022) Tight network connectivity within the SSE A-share sector, especially in times of crisis, with stronger connectivity within the financials, energy, and utilities sectors than other sectors [20]. Yu and Huang (2023), Yu et al. (2023) find that idiosyncratic conditional skewness affects stock returns within a stock sector through information channels such as predicting long-term cash flows, correlation with business conditions, and association with market participant behavior [21], dividend growth (decomposing yields into smoothed and residual components) [22]. Information flow indicates the circulation of economic, social, human, and other elements, and is less influenced by physical location and time compared to other networks, which might better reflect the intensity of regional relationships [23]. According to the 50th Statistical Report on the Development of China’s Internet by China Internet Network Information Center (CNNIC), as of January 2022, the number of global Internet users reached 4.95 billion, accounting for 62.5% of the global population; as of June 2022, the number of Chinese Internet users was 1.051 billion, and the Internet penetration rate reached 74.4%. The creation of "Internet+" has steadily turned into a significant driving force for the economic and social innovative development of many cities and urban clusters in recent years. It is evident that the Internet has become an important part of economic and social development, and it is important to construct regional information networks based on urban information flow and explore the urban cyberspace related to digital information to understand the relative position of cities in the information world and better serve the digital economic society [24].

The use of Internet information technology to investigate and explore virtual space and combine virtual information space with real geographic space has sparked fresh ideas about the spatial changes in cities. In the era of rich information data, how identifying proper quantitative indicators to quantify the information flow between cities and construct urban networks is the key to researchers’ research. Moss (2000), Townsend (2001), and Zhen (2012)examine the hierarchical system of urban networks by modeling information flow with the help of Internet network connectivity and telephone transaction volume[25–27]. Dong(2014) used the length of landline telephone conversations between localities to design an information and communication flow network to study the spatial pattern of unit flows above the county level in Jilin Province [28]. With the advent of the Internet, a considerable amount of user behavior data has been generated, which provides important data support for the measurement of regional networks [29]. For example, Pan et al. (2019) used social network analysis to explore the spatial structure network characteristics of the Chengdu-Chongqing urban agglomeration through urban Weibo check-in big data constituting the Chengdu-Chongqing population flow information network data [30]; Xiang B (2023)used TikTok cross-city check-in data to explore the Chinese information network in terms of hierarchical attributes, community size, and node centrality spatial structure [31]; Bao Z (2021) utilized the loan records of fintech borrowers to explore the spatial structure exhibited by fintech borrower data from data on demographic characteristics, credit characteristics, and current loan information [32]. Some scholars have also used behavioral data such as Weibo data, Tencent location data, and Baidu migration data to study the spatial association characteristics of information flow [33–35], however, there are data processing problems with user behavioral data in accurately measuring information intensity [36].In reality, with the widespread use of search engines, Internet users’ search activity has become a normal social behavior in the information age, which plays an essential part in the development of intercity information flows [37, 38]. Currently, scientists have started to employ Google or Baidu’s web search data to mine the evolutionary patterns and inner processes of spatial association links in urban clusters [14, 36, 39]. The search index data based on Baidu or Google effectively represent the actual behavioral intentions of people, disclose their concerns about a social issue, and may more precisely design the spatial organization structure of information flow in urban clusters [40–42].

All the above literature has laid an important foundation for this paper to study the evolutionary characteristics of information network in Guangdong, Hong Kong and Macao Greater Bay Area, but there are still three deficiencies: first, the research area of spatial correlation of information flow involves national, provincial and related city clusters, however, there are relatively few studies for Guangdong, Hong Kong and Macao, especially the caliber of Hong Kong and Macao indicators is not uniform with Guangdong Province creates greater difficulties for the study of information network in this region; second, the current information flow research is mostly based on the analysis of undirected networks, which makes it difficult to quantify the asymmetry of information linkages between cities and ignores the important differences between the reception and release of information in cities in reality; thirdly, the relationship between Internet search behavior as a typical social behavior and the spatial structure of information networks in urban clusters is still relatively little researched, and the use of a single search engine to explain the spatial structure of information networks under the influence of multiple complex factors In addition, there are limitations in utilizing a single search engine to explain the evolution of information network spatial structure under the effect of various complex elements. In addition, information flow is quickly agitated by external variables and fluctuates substantially in the short term, therefore evaluating the influencing elements of an information network can disclose the motives behind its changes, which is vital for cognizing the evolution mechanism of network structure. Given this, this paper integrates the dual data search indicators of Google Trends and Baidu Index to overcome the weakness of single search engine data interpretation in previous studies, examines the spatial association characteristics and evolution patterns of information linkages in Guangdong-Hong Kong-Macao Greater Bay Area, and quantifies the node characteristics reflected by asymmetric linkages in the information network. At the same time, we evaluate what factors influence the "gradient" of unbalanced information linkages in Guangdong, Hong Kong, and Macao based on QAP analysis, to give a scientific basis for the joint growth of Guangdong, Hong Kong, and Macao urban agglomerations.

### Data and methods

#### Study area

The Guangdong-Hong Kong-Macao Greater Bay Area is located in the coastal region of South China (111°21′~114°53′E, 21°28′~24°29′N) and consists of two special administrative regions, Hong Kong and Macau, and nine PRD cities in Guangdong Province, namely Guangzhou, Shenzhen, Zhuhai, Huizhou, Dongguan, Zhongshan, Jiangmen, Foshan, and Zhaoqing. Foshan and Zhaoqing are two of the nine PRD cities in Guangdong Province. The region has exceptional geographical circumstances, is "surrounded by mountains on three sides, three rivers converge," has a long coastline, a good port group, a vast sea surface, and is a location rich in light, heat, and water resources in the country. As an important spatial carrier for the country to build an international science and technology innovation center, the Guangdong-Hong Kong-Macao Greater Bay Area has a good endowment of innovation resources, unique innovation vitality, an open innovation environment, a globalized innovation pattern, and a deep foundation of regional science and technology innovation collaboration, It is the world’s gathering point for regional innovation and has traditionally been one of the most open and economically dynamic regions in China [43]. By the end of 2021, the regional resident population will be 86,692,300, accounting for 6.12% of the total national population. The region achieves a total GDP of RMB 12.6 trillion in 2021, accounting for nearly 11% of the entire national GDP.

#### Data sources

Taking into account the popularity and development of Internet information and search engines, the relative stability of information networks over time, and the accessibility and comparability of relevant data, this paper selects Baidu Index (http://index.baidu.com) and Google Trends (trends.google.com/) as research data based on the actual situation of engine usage in Guangdong, Hong Kong, and Macau. Baidu Index and Google Trends are key statistical analysis platforms in the contemporary Internet big data era based on the huge Internet user behavior data of Baidu and Google. The search volume of Internet users on Baidu web pages and Google homepages is used as the database of the search index, and the weighted sum of the search frequency of specific keywords on engine web pages is analyzed and calculated to reflect the information access of residents in a certain area for a certain thing in a certain period of time.

The search data in the engine is obtained as follows: In this paper, using the keywords of 11 cities in the Guangdong, Hong Kong and Macao Greater Bay Area, the daily average search volume in the "Comparative Analysis of Cities" of the Baidu Index search interface in Baidu Browser, and the average of city-specific searches of search items in Google Trends in Google Chrome Browser were used to characterize the search data, thus measuring the information connection between the cities. Based on the information flow matrix connecting cities from 2012 to 2021, the structural characteristics and evolutionary patterns of the information network of cities in the Guangdong-Hong Kong-Macao Greater Bay Area are investigated.

In the analysis of the influencing factors part, due to the strong impact of external environmental factors in 2021, in order to avoid the influence of external interference on the analysis results, the above data are used 2018 data results, where the city distance mileage results are obtained through Baidu map tool query, and the rest of the data are obtained from the Guangdong Provincial Statistical Yearbook, China Statistical Yearbook, Hong Kong Yearbook and Macau Yearbook, where, the R&D science and technology R&D expenditure of Hong Kong and Macao is calculated according to the calculation method of mainland science and technology R&D expenditure, which is obtained by multiplying the 2018 GDP of Hong Kong and Macao cities by the 2018 R&D expenditure input intensity of Hong Kong and Macao.

#### Methods

The term "social network" refers to a collection of social actors and their relationships, expressed in terms of points and lines, i.e., a social network is a collection of multiple nodes and lines between them, with the nodes representing individual actors in a social group and the lines in between representing the relationships between actors (2004).

The social network analysis (SNA) method stresses the attention on the "relationships" of the data, and the structural aspects of the various "relationships" are investigated from a quantitative perspective. By loading the data into Ucinet 6.0 and doing social network analysis, the network topology may be determined. In this research, we use the search engine to develop the information link strength matrix as a quantitative basis to examine the information linkage in the Guangdong-Hong Kong-Macao Greater Bay Area.

### Centrality analysis

Centrality reflects the size and centrality of the "power" of a node in the network and the degree of dependency of other cities on it, which is a quantitative measurement of the power of a single node. With reference to the real scenario of information connection among node cities, Degree Centrality and Eigenvector Centrality are selected as representative indicators in this work.

### Degree centrality

The degree centrality of a point represents the degree to which the node is at the core of the network, and the bigger the number of nodes directly connected to the node city, the greater the degree centrality of the point. Degree centrality is used to describe the direct link of a node to other nodes, including point-in and point-out degrees [44]. The point-in degree represents the ability of a node city to receive information connections from other cities; the point-out degree indicates the ability of a node city to send information connections to other cities. The equations are respectively:

$$C_{ei}=\frac{\sum_{j=1,j\neq i}^{n}l_{ij}}{n-1};C_{eo}=\frac{\sum_{j=1,j\neq i}^{n}l_{ij}}{n-1}$$ (1)

where *l*_*ij*_ and *l*_*ji*_ describe whether there is a direct information connection between two nodes i(j) and j(i) cities, respectively, and *n* denotes the number of city nodes in the network.

#### Eigenvector centrality

The eigenvector centrality can also visualize the influence of nodes in the information network. The power of a node city depends not only on the number of neighboring relationships but also on the quality of those relationships, i.e., on the importance of the neighboring cities. The eigenvector centrality measures the "quality" of information connections between neighboring cities, which is given by the following formula:

$${\text{EC(i)}=\text{x}}_{\text{i}}=\text{c}\sum_{\text{j}=1}^{\text{n}}{\text{a}}_{\text{ij}}{\text{x}}_{\text{j}}$$ (2)

Where *c* is the proportionality constant and *x* is a measure of the importance of the information network, denoted *x* = [*x*_1_,*x*_2_,*x*_3_,…,*x*_*n*_]^*T*^.

#### Core-edge structures analysis

Core-edge structure refers to the spatial structure of a network with a tight core and dispersed edges that are organically composed of several nodes. The core area has a superior geopolitical location, can access more network resources, and plays a leading role in regional spatial development, while the development and planning of the edge areas usually depend on the core area’s drive. The boundary delineation and spatial relationship between the core and peripheral areas are constantly adjusted and dynamically changed. The core-edge structure analysis of social network analysis provides a practical and quantitative expression of the "core-edge" theory of spatial economics through the measurement of the "nucleus" of nodes, which can clarify the specific position of the urban innovation ecosystem in the overall network. The larger the number of nuclei in a city, the closer it is to the core of the network, and the greater its network dominance. The core-edge analysis can provide a scientific basis for strengthening the collaborative innovation development of the Guangdong-Hong Kong-Macao Greater Bay Area city cluster, and also contribute theoretical references for the optimization of the spatial structure of the regional innovation ecosystem. The core-edge model can be divided into the continuous "core, semi-core, edge" structure and the discrete "core-edge" structure, depending on the selection of fixed ratio data or fixed class data.

#### QAP analysis method

In social network analysis, the QAP analysis approach based on the permutation of matrix data can overcome the inadequacies of multiple regression analysis due to the correlation of the data itself, which violates the principle of multicollinearity. QAP analysis can be used to compare the correlations of elements in two matrices to determine the correlation coefficients between the two matrices, including QAP correlation analysis and QAP regression analysis [45]. The steps are as follows: first, calculate the correlation coefficients of the two matrices; second, repeat at least hundreds of random permutations on the rows and columns of one of the matrices to obtain the entire correlation coefficient distribution; third, compare the correlation coefficients of the actual matrix obtained in the first step with the distribution of the correlation coefficients obtained by random permutations in the second step, and analyze whether the correlation coefficients of the actual matrix fall in the acceptance domain or the rejection domain, to The conclusion of the analysis is achieved.

In this paper, using the QAP analysis method, we take the strength of information linkage among Guangdong, Hong Kong, and Macau cities as the dependent variable, and determine the independent variables within the scope of demographic factors, urban economic development, urban distance, information infrastructure construction, science and technology education capacity, etc. After examining the comprehensive circumstances, searching databases, and statistical yearbooks, we decided to select The number of employed people, urbanization rate, straight-line distance to the city, number of cell phone subscribers, and R&D research money were selected as dependent variables. The model is constructed as follows:

F=f(P,E,D,I,S) (3)

Where *P* represents the demographic factor, expressed as a matrix of differences in the number of employed people; *E* represents the economical development of cities, expressed as a matrix of differences in the urbanization rate; *D* represents the distance of cities, expressed as a matrix of linear kilometers between cities; *I* represents the information infrastructure development, expressed as a matrix of differences in the number of cell phone subscribers between cities; *S* represents the education and research situation, expressed as a matrix of differences in R&D research funding between cities.

## Results and analysis

### Characterization of information networks

#### Results of information linkage strength analysis

**Fig 1:**
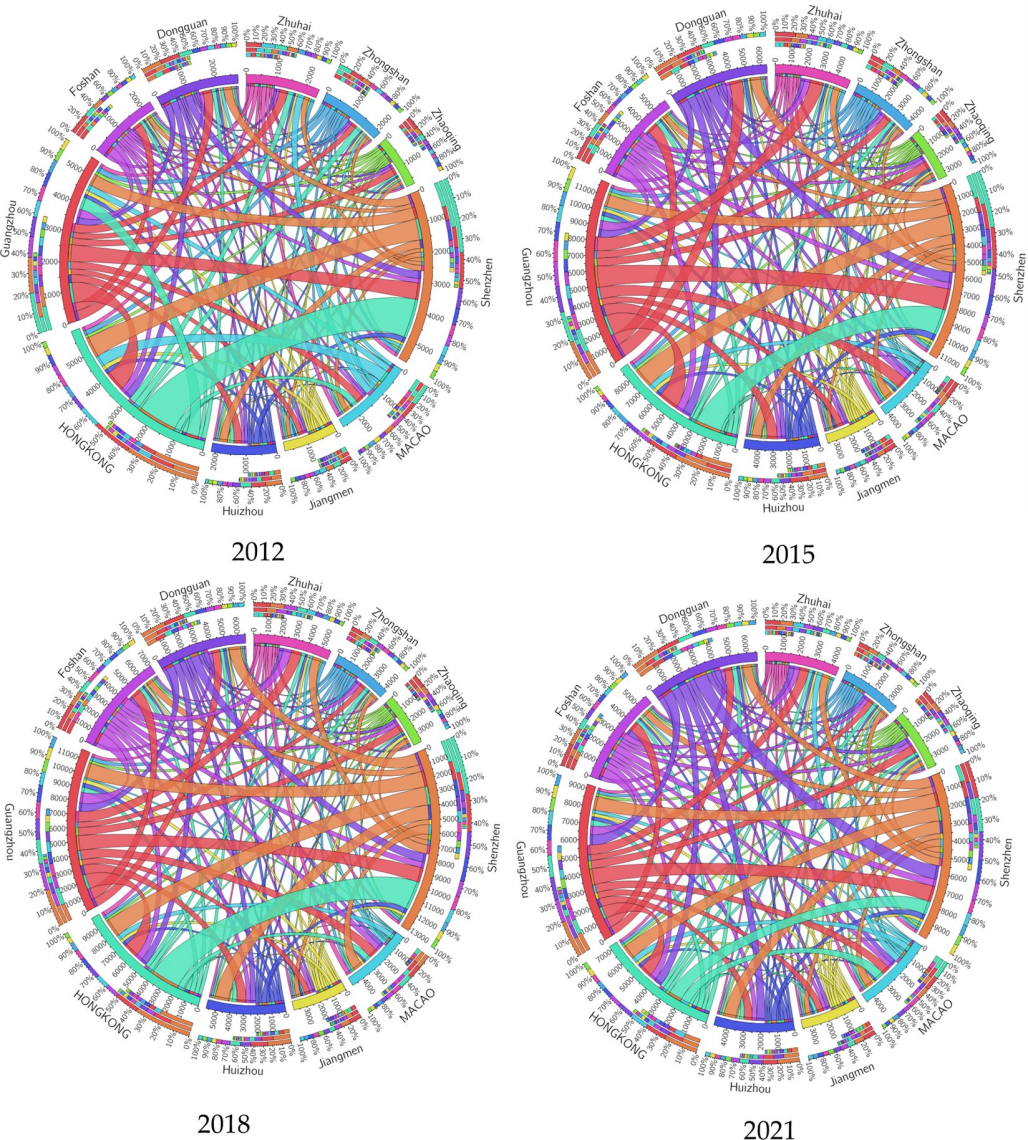
Visualization structure of the change of information linkage intensity in the Guangdong-Hong Kong-Macao Greater Bay Area city cluster, 2012–2021.

To visualize the structural characteristics and evolution law of the information network of the Guangdong-Hong Kong-Macao Bay Area city cluster, this paper constructs a visual structure diagram of the change of information connection strength in the Guangdong-Hong Kong-Macao Bay Area through ArcGIS geographic analysis software. As illustrated in Fig 1., each node represents a distinct city, and the information connection between cities is represented by the line segments between each node, and the thickness of the line segments shows the strength of the information connection between cities.

From the above **Fig 1.**, we can intuitively see that the information linkage strength network of the Guangdong-Hong Kong-Macao Greater Bay Area city cluster as a whole presents a triangular pattern dominated by three nodes: Guangzhou, Shenzhen, and Hong Kong, which has been evolving dynamically around the three core cities of Guangzhou, Shenzhen, and Hong Kong during 2012–2021. In 2012, only Shenzhen and Hong Kong had an information link strength value of above 700, which is substantially greater than the other cities. The strength of inter-city links is substantially higher than that of the rest of the cities. Compared with 2012, the information link strength of the city cluster in 2015 is greatly strengthened, and the intra-regional links are closer, with Dongguan joining the Guangzhou-Shenzhen-Hong Kong dominant city circle. At the same time, Guangzhou-Foshan, Guangzhou-Zhuhai, Guangzhou-Macao, and Guangzhou-Huizhou city information linkage intensity values exceed 700, entering the ranks of high-intensity city linkage, with Guangzhou as the leading secondary linkage city circle initially formed, and the city information network is in a rapid development stage. In 2018, the remaining cities and the leading city linkage intensity increased significantly: "Guangzhou, Shenzhen, Hong Kong, Dongguan, Huizhou, and Zhuhai." In 2021, under the combined impact of the external environment and economy, the information connectivity among cities in Guangdong, Hong Kong, and Macao will be more and more powerful. In 2021, under the combined impact of the external environment and economy, the strength of information linkage among cities in the Greater Bay Area weakens, the value of linkage strength among cities decreases to different degrees, and the overall network sparseness is slightly lower than the level in 2015; overall, except for the strong information linkage among Guangzhou-Shenzhen-Hong Kong, only Shenzhen-Huizhou and Shenzhen-Dongguan information linkage strength values remain high above 700, which are less affected by the external impact. From a comprehensive perspective, the information linkage intensity of the Guangdong-Hong Kong-Macao Greater Bay Area shows a trend of gradual increase of radiation influence from the leading cities of Guangzhou-Shenzhen-Hong Kong to the surrounding cities during the period of 2012–2021, and a closer and more balanced high-intensity network of information linkage in the Guangdong-Hong Kong-Macao Greater Bay Area is gradually formed.

### Results of the centrality analysis

By assessing the degree centrality of the information connection strength of each city in the Guangdong-Hong Kong-Macao Greater Bay Area city cluster (Table 1), it can be seen that each city has considerably improved both in terms of output and attractiveness during the decade of 2012–2021. In terms of point-out degree, over the years, Guangzhou has always maintained the first place in terms of out-degree, which is much higher than its in-degree, but the relative value of its in-degree gap has gradually shrunk. The next higher degree cities are mainly Hong Kong and Shenzhen; Shenzhen pointed out degree value in 2015 after overtaking Hong Kong for many years to maintain the second place in the Greater Bay Area city group; Foshan and Dongguan cities followed, demonstrating the rapid growth of information linkage output during the study period. In terms of point-in degree, Hong Kong and Macau have maintained a higher level in the Guangdong-Hong Kong-Macao Greater Bay Area for many years, and from 2012 to 2015, Guangzhou and Shenzhen have significantly increased the amount of information attracted, with Shenzhen jumping and remaining in the first place in the Guangdong-Hong Kong-Macao Greater Bay Area in 2015, followed by Hong Kong and Guangzhou, and Zhuhai surpassing Macau and ranking fourth in the Bay Area in terms of information attraction since 2015. In summary, with its total control advantage in regional information linkage, Guangzhou-Shenzhen has become the extreme center of information exchange in the Guangdong-Hong Kong-Macao Greater Bay Area, with a high information exchange level and dispersion influence. Foshan, Dongguan, Zhuhai, and Macao have significant talents in information export and attraction, respectively, and these four cities have the radiation intensity and transit impact to become the regional information connection center. Overall, the regional information linkage of the Guangdong-Hong Kong-Macao Greater Bay Area presents the characteristics of low exchange threshold, low-cost hindrance, and rapid response due to the influence of rapid technological development, which makes the city system downscale and the overall information linkage network of the city cluster tend to be structurally flat while showing the characteristics of polycentricity.

**Table 1:** The degree centrality of cities in the Guangdong-Hong Kong-Macao Greater Bay Area.

Years Cities	2012		2015		2018		2021	
	out-degree	In-degree	out-degree	In-degree	out-degree	In-degree	out-degree	In-degree
HongKong	2965	2937	3456	5456	4760	5126	3448	3781
Macao	1236	2934	1146	3086	1267	3632	1659	2891
Guangzhou	3025	933	8242	3597	7218	4397	5677	3559
Shenzhen	2428	1261	5696	5628	7549	5638	5502	4359
Zhuhai	841	2092	1742	3166	1938	3704	1486	2928
Foshan	1204	985	3535	2380	4312	2740	3273	2398
Dongguan	1289	823	3503	2874	3649	2891	4247	2360
Huizhou	852	1288	2131	2732	2117	3569	1748	3012
Zhongshan	1003	894	2130	2166	2026	2192	1602	1993
Zhaoqing	663	1220	1368	1966	1327	2404	1280	2304
Jiangmen	816	955	1824	1722	2035	1905	1620	1957

The findings of the information linkage of the eigenvector centrality and normalized eigenvector centrality measures of cities in the Guangdong-Hong Kong-Macao Bay Area derived by the UCINET metric are displayed in Table 2. In 2012, the importance of the remaining cities in the Bay Area was much lower than that of Guangzhou-Shenzhen-Hong Kong, indicating that there was a certain polarization phenomenon within the Bay Area city cluster, and the advantages of resource elements were concentrated in Guangzhou-Shenzhen-Hong Kong, while the centrality of Guangzhou was slightly weaker than that of Shenzhen and Hong Kong, indicating that In 2015, the centrality of Foshan, Dongguan, Huizhou and other cities adjacent to Guangzhou showed a significant increase in the Eigenvector centrality, and Guangzhou’s centrality quickly rose to the first place, indicating that Guangzhou had a significant positive radiation influence on the surrounding cities and received positive feedback. after 2018, Shenzhen and Guangzhou will be in the hub of the city cluster, and spillovers to other cities. The influence drives other cities in the Bay Area to increase their centrality significantly, and the enhancement of information links among other cities in the Bay Area diminishes the problem of differences within the Bay Area city cluster, which also indicates that the information link strength network in the Guangdong-Hong Kong-Macao Greater Bay Area goes flat and multipolar, and the city cluster shows a good and balanced development trend within the city cluster. It is worth noting that in 2021, Dongguan overtakes Hong Kong and becomes the third center behind Guangzhou and Shenzhen. It is apparent that Dongguan, with its geographical location advantage, fully absorbs the spillover influence from the two key cities of Guangzhou and Shenzhen and becomes another prospective metropolis in the Guangdong-Hong Kong-Macao Greater Bay Area that cannot be overlooked. Macau is the only city with decreasing importance in the lower eigenvector centroid value besides the central cities and needs to actively integrate its resource advantages and strengthen communication with other cities in the Bay Area to avoid the risk of marginalization, which should be focused on and supported in regional cooperation.

**Table 2:** The Eigenvector centrality of cities in the Guangdong-Hong Kong-Macao Greater Bay Area.

Years Cities	2012		2015		2018		2021	
	Eigenvector	nEigenvecor	Eigenvector	nEigenvecor	Eigenvector	nEigenvecor	Eigenvector	nEigenvecor
HongKong	0.537	76.003	0.426	60.275	0.423	59.824	0.322	45.579
Macao	0.224	31.733	0.229	32.392	0.240	33.871	0.221	31.195
Guangzhou	0.428	60.583	0.493	69.653	0.422	59.676	0.446	63.023
Shenzhen	0.515	72.825	0.460	64.992	0.497	70.240	0.457	64.599
Zhuhai	0.196	27.720	0.230	32.494	0.246	34.790	0.239	33.735
Foshan	0.174	24.646	0.246	34.798	0.265	37.474	0.279	39.493
Dongguan	0.222	31.436	0.279	39.474	0.259	36.557	0.353	49.959
Huizhou	0.184	26.083	0.222	31.407	0.259	36.625	0.276	39.039
Zhongshan	0.156	22.101	0.167	23.638	0.161	22.781	0.176	24.889
Zhaoqing	0.133	18.879	0.150	21.164	0.162	22.853	0.201	28.413
Jiangmen	0.133	18.805	0.146	20.596	0.149	21.099	0.182	25.694

### Results of core-edge analysis

To further identify the structure and changes of the information linkage network of each city in the Guangdong-Hong Kong-Macao Greater Bay Area city cluster, this paper constructs a continuous spatial core-semi-core-edge model of the information linkage strength of the Guangdong-Hong Kong-Macao Greater Bay Area, as shown in Table 3 from 2012 to 2018, Shenzhen and Hong Kong always occupied the position of the core area, Guangzhou was among the core area in 2015, Guangzhou entered the core area, and under the radiation influence of the core cities, the range of semi-core cities has been expanding, and mainly concentrated with the cities adjacent to the core area. With the geographical location advantage and policy dividend, Foshan, Dongguan, Huizhou, and Zhuhai not only directly enjoy the positive external influence of the diffusion effect of the core region, but also enjoy the advantages of low cost and high efficiency of information linkage, Foshan, Dongguan, and Huizhou became semi-core cities in 2015, and Zhuhai joined the semi-core region in 2018. 2021, to the impact of external economic environment variables, coupled with the variations among Guangdong, Hong Kong, and Macao institutions and regulations, Hong Kong’s core city position slides to semi-core, and Dongguan leaps into the spatial center of the Guangdong-Hong Kong-Macao Greater Bay Area. Overall, the four cities of Guangzhou, Dongguan, Shenzhen, and Hong Kong represent the core city region of the Guangdong-Hong Kong-Macao Greater Bay Area in the northwest-southeast direction and gradually exert the radiation spreading effect to the northeast-southwest. The "core, semi-core, edge" pattern is clearly defined. Zhongshan and Macau have geographical advantages, but have not yet completely released their information exchange potential and need to further enhance their advantages to actively receive the spillover impacts of the core area. Jiangmen, Zhaoqing, and other regions are geographically constrained from receiving the radiation influence of the core urban areas and need to make efforts to overcome the geographical disadvantages, improve their information attraction and input capacity according to local conditions, and promote the formation of a high-intensity, multi-center linkage information intensity network structure.

**Table 3:** Spatial core—semi-core—edge structure of information linkage intensity in Guangdong-Hong Kong-Macao Greater Bay Area urban agglomerations, 2012–2021.

Year	Core cities	Semi-core cities	Edge cities
2012	Shenzhen、HongKong	Guangzhou、Dongguan	Foshan、Huizhou、Zhongshan、Zhuhai、Macao、Jiangmen、Zhaoqing
2015	Guangzhou、Shenzhen、HongKong	Dongguan、Foshan、Huizhou	Zhongshan、Zhuhai、Macao、Jiangmen、Zhaoqing
2018	Guangzhou、Shenzhen、HongKong	Dongguan、Foshan、Huizhou、Zhuhai、Macao	Zhongshan、Jiangmen、Zhaoqing
2021	Guangzhou、Shenzhen、Dongguan	HongKong、Foshan、Huizhou、Zhuhai、Macao	Zhongshan、Jiangmen、Zhaoqing

### Results of the analysis of factors influencing information linkages in the Guangdong-Hong Kong-Macao Greater Bay Area city cluster

Information flows such as mail volume, telecommunication services, Internet traffic, and transportation flows comprising road, train, and port throughput are significant drivers that shape city network relationships [42], and city networks adapt and develop under the impact of numerous variables. From the perspective of traditional city information linkage, the difference in population and other factors contribute to the difference in demand for information exchange between cities; the level of technology and education; the information infrastructure; and other factors make information exchange different and more efficient; the distance of city transportation and other factors influence the relationship and the way of exchanging resources and products between cities, and many factors contribute to the formation of an intricate network characteristic of the information linkage network. According to current research findings, the following variables have been identified as influencing factors.

Influencing factor selection

### Demographic factors

Here the population factor is not all resident population data, the traditional city size class and the number of the year-end resident population are closely related, but this as a measure of the larger population base includes the elderly population, preschool population, etc.; the correlation between these populations and engine search data is weak, and the entire population can only represent the upper limit of external information contact under the full penetration of the Internet. Population movement is a big element of our country. The fundamental reason for population mobility, is population migration for jobs and job hunting. The number, character, and distribution of employed people are intimately tied to the level of economic growth of a country or region. In this sense, Guangdong is a big province with population mobility, High levels of economic development in the province, urban employment, entrepreneurship, and other opportunities, attracting inflows of the population from cities with low levels of economic development, which leads to improved health care, education, and other aspects, as well as additional resources to the Convergence of resources to densely populated areas [46, 47]. The search of the target city by the employed people is an important technique to collect relevant information, that is, to have an impact on the information linkage data.

#### City economic development

The level of economic development in a city typically impacts the evolution of the information network in the region. The level of economic development of a region determines the construction of local infrastructure, the demand for employment development, the quality of life of the people, the purchasing power, and the level of investment in information network infrastructure, which is the basis for the development of each city. Cities with a relatively high level of urban economic development have created a favorable environment for Internet development, and the network’s strong innovation vitality has allowed the network to rapidly integrate into various scenes of people’s lives and form new business and service models, while the vast application scenes and consumer demand have also forced enterprises to constantly learn new technologies, raising the bar even higher [48].

#### City transportation distance

Inter-city distance and accessibility are the basis for the mutual interchange of population and goods between two cities. The proximity of cities has a substantial influence on the economic exchanges between cities and is also an essential influence on the exchange of resources, culture, knowledge, and other elements between cities. The closer the distance between two cities and the better the transportation infrastructure, the more advantageous the transit convenience, commuting time, and the cost of the inter-city flow of various factors, and the more frequent the inter-city exchange of various factors. From the internal perspective of the Bay Area, such as Guangzhou and Foshan, Zhuhai and Macao, Shenzhen and Dongguan, due to the proximity of the cities, shorter commuting time, affected by the differences in regional housing prices, ease of living, etc. some workers choose to work in the local area and live in other places of life, and the mutual exchange of various elements is closer than in other cities, resulting in the formation of a denser information network link [49].

### Information infrastructure construction

The level of information infrastructure construction plays a significant role in the growth of an information linkage network. In the age of information development, information products, and electronic devices have become an integral aspect of personal life and business. The development and enhancement of regional information infrastructure, the increase of cell phones, broadband users, etc., the popularization of the Internet, broadband rates, the expansion of rich network services, and more convenient contact between people and cities. New information infrastructure is represented by 5G, the Internet of Things, gigabit fiber, and the Internet. In the context of the current era of development, high-quality information infrastructure can provide better support for the digital transformation of urban enterprises. At the same time, it can provide high-quality information services for urban users, which tends to promote the development of inter-city information networks in the direction of closer and higher agglomeration, further releasing the potential of information consumption and promoting economic and social development.

### Science and technology education

In the era of science and technology information, the status of science and technology education in all aspects of urban development should not be ignored. From the perspective of the Greater Bay Area, the development of science and technology innovation and talent training are important elements of the development of the Bay Area, and the demand for network applications and changes in information technology are the frontier elements of science and technology development. Personal information network material is demanded by their cultural literacy, education level, tastes, and other more subjective factors (personal literacy, technology level, etc.), as well as the surrounding area’s information connection efficiency, and so on. High-quality talents continue to gather in areas with strong research capabilities, higher demand for technology products and other products, higher acceptance of the frequency of network technology updates, easier to master high-quality technology, driving demand and promoting more efficient development of information technology, etc [50].

Descriptive statistics of the data results for the selected representative indicators are shown in Table 4.

**Table 4:** Descriptive statistics of variables.

Variable	Obs	Mean	Std. dev.	Min	Max
Number of employed people	55	367.7876	276.6802	12.31	1011.71
Urbanization rate	55	18.992	15.24651	0	53.01
Distance to city	55	1228.302	896.3887	30.69	3122.33
Number of cell phone subscribers	55	405.5025	364.7593	1.51	1139.21
R&D research funds	55	92.0127	40.89789	12.6	199.8

#### Results of the empirical analysis of the factors influencing the characteristics of the urban information linkage network

In this paper, QAP correlation and regression analysis methods are selected to empirically analyze the correlations and regression relationships that exist between the explanatory variables of the information link strength matrix and the five explanatory variables consisting of population, science and technology, and transportation.

#### Results of QAP correlation analysis

Using Ucinet software, the information linkage intensity matrix of the Guangdong-Hong Kong-Macao Bay Area city cluster and the derived five influencing factors matrix were evaluated one by one, and the results of the QAP correlation analysis are displayed in Table 5.

**Table 5:** Results of QAP Correlation Analysis on the Influencing Factors of Information Linkage Network in Guangdong, Hong Kong, and Macao Greater Bay Area.

Indicators	Actual correlation coefficient	Significance level	Mean value of correlation coefficient	Standard deviation
Number of employed people	0.252	0.034	0.001	0.111
Urbanization rate	0.124	0.028	0.000	0.068
Distance to city	-0.227	0.000	-0.001	0.096
Number of cell phone subscribers	0.184	0.087	0.001	0.108
R&D research funds	0.256	0.059	-0.001	0.102

It can be seen that employment population, city economic development, city distance, information infrastructure construction, and science and technology education capacity all have significant effects on information linkage strength, among which, city distance matrix D has a negative correlation with information linkage strength matrix R. The correlation coefficient between R and D is -0.227, which demonstrates that distance cost has a considerable inhibiting influence on information linkage strength; that is, the closer the distance between two cities, the greater the information linkage. The negative association between the two also reveals, to a certain extent, the influence of traffic accessibility on information link strength, indicating that improving traffic accessibility has a bigger function in fostering inter-city connectivity. The information linkage strength matrix R shows positive correlations with several variables P, E, I, and S, with correlation coefficients of 0.252, 0.124, 0.184, and 0.256, respectively, indicating that population size, urban economic development, information infrastructure construction, and science and technology education capability have positive effects on inter-city information linkage strength. With the increase in the employed population, the scale of the urban population is expanding, the basic needs such as living are growing, and the continuous improvement of the living environment will attract more population, pushing the further expansion of the urban population scale and prompting the increasing information concern of cities. The expansion in the urban economic development level draws the investment and construction of firms in other regions, and the rise in intra-city demand will boost trade and cooperation with other regions. The improvement of information infrastructure construction to promote the continuous development of urban informatization, the enhancement of science and technology education to promote the cultivation of high-quality talents in science and technology, information technology research and development capabilities, and inter-city information links will be enhanced.

Science and technology education has the biggest influence on the level of information connection among the five contributing indicators. The employed population and the distance between cities are both relatively large, indicating that the distance between cities has a significant impact on information linkage between cities and that the spatial cost of transportation, to some extent, inhibits the rapid development of the information linkage network of the entire Guangdong-Hong Kong-Macao Greater Bay Area city cluster. Some appropriate actions should be taken, such as upgrading the mode of transportation between two cities and building and improving the efficient transportation system of the entire city cluster. The development and enhancement of the network system can improve the spatial structure of the information network. The influence of the level of urban economic development and information infrastructure on the strength of the information network is relatively weak, owing to the fact that the overall Internet penetration rate and the number of broadband users in the Guangdong-Hong Kong-Macao urban agglomeration have been rapidly rising in recent years due to the popularization and massive use of broadband Internet and have always remained at the forefront of the country, which has had a certitude Regions with a high level of economic development have a larger penetration rate of electronic gadgets, the Internet, and so on, as well as more perfect information infrastructure building. These two variables have rather substantial relationships, promoting the evolution and development of information network connections.

### Results of QAP regression analysis

The findings of the regression analysis are reported in Table 6. From the data results, the five indicators picked have strong significance, and all five indicators passed the 1% significance test. The standardized regression coefficient of population size (P) is 1.45, which suggests that the population component has a stronger impact on the strength of information linkage when numerous factors are combined. The bigger the number of employed people, the more they drive the development of diverse industries and employment, and the more frequent the information connectivity among cities tends to be, which has a more direct impact on the strength of information linkage. The standardized regression coefficient of city economic development level (E) is 0.245, which suggests that the level of economic development of cities in the Guangdong-Hong Kong-Macao Greater Bay Area is closely related to the strength of inter-city information connectivity. The standardized regression coefficient of city distance (D) is -0.134, suggesting that city distance has a substantial influence on the construction and evolution of the network of city information link strength, and the closer the geographic areas are, the closer the information links are. The role of distance and transportation variables cannot be overlooked in constructing city information links, but the presence of other elements, the existence of diverse conditions such as city scale, would to some extent weaken the information links. The inhibiting effect of city distance on the growth of an information network.

**Table 6:** Results of QAP regression analysis of the factors influencing the information linkage network of Guangdong, Hong Kong and Macao Greater Bay Area.

Indicators	Unstandardized regression coefficient	Standardized regression coefficient	Significance probability	P≥0	P≤0
**Intercept**	102946.953125	0.000000			
Number of employed people	869.091614	1.444842	0.001	0.001	1.000
Urbanization rate	2160.118652	0.245122	0.001	0.999	0.001
Distance to city	-145.292099	-0.133892	0.004	0.996	0.004
Number of cell phone subscribers	244.963181	1.425023	0.000	1.000	0.000
R&D research funds	0.277252	0.482279	0.000	0.000	1.000

The standardized regression coefficient of information infrastructure construction (I) is 1.43, which indicates that in a state where multiple influencing factors coexist, information infrastructure construction plays an extremely important role in the strength of information network linkage, and the continuous improvement of information infrastructure will continue to promote closer inter-city information linkage and the changing development of the information linkage network of the whole urban cluster. The regression coefficient of scientific research and education (S) is 0.48, which indicates that the development of an information network is closely linked with education and scientific research factors, which enlightens us that in the process of the evolution of inter-city information linkage network, in the construction of Guangdong-Hong Kong-Macao Greater Bay Area, sufficient investment in scientific research, promoting scientific research and education, and the improvement of information and scientific research technology will help improve the quality of regional personnel themselves and promote the way of information linkage, efficiency, and other aspects. The transformation and development of information linkage will have a clear driving effect on the city’s information linkage.

## Conclusions

### Research conclusion

In the era of big data and information, the application and growth of digital information technology have enveloped the whole society with highly developed communication networks. The urban network constituted of information flow has become an important direction for scientists to examine the spatial pattern of urban agglomerations. In this paper, using Baidu and Google search data as indicators, UCINET software, and social network analysis methods, we constructed the information connection strength network of 11 cities in the Guangdong-Hong Kong-Macao Greater Bay Area city cluster and conducted a comparative analysis of the development and evolution characteristics of the overall city cluster information network in the region from 2012–2021 and its five influencing factors.

First, from the results of the analysis of information linkage intensity characteristics, the influence and influence degree of each city have increased substantially during 2012–2021; the degree of mutual influence among the dominant city groups is relatively greater, and the peripheral cities are controlled by the dominant city groups to a greater extent, and the overall information linkage intensity of the Guangdong-Hong Kong-Macao Greater Bay Area shows the gradual increase of radiation influence from the dominant city of Guangzhou-Shenzhen-Hong Kong to the surrounding cities. In 2018, the six cities of Guangzhou, Shenzhen, Hong Kong, Dongguan, Huizhou, and Zhujiang established a high-intensity information linkage city circle, and a closer and more balanced high-intensity network of information linkage in the Guangdong-Hong Kong-Macao Greater Bay Area is progressively formed.

Secondly, from the analysis of centrality characteristics, the degree centrality and feature vector centrality of Guangdong, Shenzhen, and Hong Kong are both in the high position of the cities in the Greater Bay Area, and they have the absolute control advantage in the regional information linkage, with high information exchange level and diffusion influence. The four cities of Foshan, Dongguan, Zhuhai, and Macau have the radiation intensity and transit effect to become the regional information linkage hub. Due to the rapid development of technology in the Guangdong-Hong Kong-Macao Greater Bay Area, the regional information linkage is characterized by a low exchange threshold, low-cost hindrance, and rapid response, which makes the city system downscale and the overall information linkage network of the city cluster tend to be structurally flat and polycentric at the same time. Macau is at risk of being isolated and needs to actively integrate its resource advantages, increase communication with neighboring cities in the Bay Area, governments should provide focused attention and policy support in regional cooperation.

Third, from the results of the core-edge study, the core node cities have extraordinarily strong control over the surrounding cities, and the siphoning impact is extraordinary. In recent years, the range of semi-core cities has been increasing under the full radiation impact of the core cities, and the four cities of Florida, Dongguan, Huizhou, and Zhujiang are developing swiftly with their geographical advantages and policy rewards. The four cities of Guangzhou, Dongguan, Shenzhen, and Hong Kong constitute the core city area of the Guangdong-Hong Kong-Macao Greater Bay Area in the northwest-southeast direction and gradually exert the radiation diffusion effect to the northeast-southwest, with the core-semi-core pattern clearly defined. Zhongshan, Macao, Jiangmen, Zhaoqing, and other regions need to improve their information attraction and input capacity according to their local conditions to help the formation of a high-intensity, multi-center linkage and the balanced development of the information linkage network in the Guangdong-Hong Kong-Macao Greater Bay Area.

Fourth, according to the results of the QAP analysis, the expansion of the employment population, the improvement of urban economic development level, the proximity of cities, the improvement of transportation accessibility, the improvement of urban information infrastructure construction, and the improvement of scientific and technological capability quality all contribute to the closer information linkage network of urban clusters.

### Policy recommendations

The engine search volume as one of the linkage indicators representing the huge information linkage system, mainly to study the attention of users within the city to other cities, and constitute the linkage network, can not fully represent all the inter-city information network linkage, networked big data development trend, Baidu, Google as the main search engine for domestic and international Internet users, to Baidu index, Google trends to study the information linkage The network tends to be more accurate and authoritative. The information linkage network of the Guangdong-Hong Kong-Macao Bay Area displays an imbalanced development, and there is a danger that the neighboring cities are ignored. The information network of the Bay Area cities needs to be streamlined and updated to better assist the creation of the world-class city cluster in the Guangdong-Hong Kong-Macao Bay Area. Based on the foregoing conclusions, this study provides the following recommendations:

First, based on infrastructure construction, promote the integration of information infrastructure in the Bay Area. The "Internet Plus," cloud computing, and artificial intelligence, as new information infrastructure, penetrate numerous disciplines and lead the frontier of research and technology. Combining with the development needs of the digital economy and the development strategy of the Guangdong-Hong Kong-Macao Bay Area, it is necessary to support the transformation and upgrading of traditional infrastructure within city clusters, promote the interconnection and interoperability of information infrastructure, improve network security protection mechanisms, and realize cross-regional common construction and sharing of important facilities, provide a basic guarantee for integrating resources and elements of city clusters, promote the coordinated development of cities in the Bay Area, ensure efficient interaction within city clusters, promote the construction of a smart Bay Area, etc., and promote the overall comprehensive integration process of the Guangdong-Hong Kong-Macao Greater Bay Area with high efficiency.

Second, give full play to the role of talent policy to attract high-quality people and promote science and technology education. To alleviate the phenomenon of widening the gap between cities due to the concentration of high-quality talents and high-level scientific research and education, each city within the city cluster should base itself on itself, attract scientific and technological talents in a targeted manner through industrial science and technology parks, scientific and technological talent policies, etc., promote professional scientific and technological innovation, and develop development strategies by taking advantage of their fields according to local conditions. The peripheral areas should actively play the advantage of resources, take the initiative to establish complementary exchanges and cooperation with neighboring cities and semi-core cities, drive their development while promoting the optimal allocation of resources within the Bay Area, and gradually form a regional city "group" collaboration, multi-group cooperation of the city linkage dynamic development evolution pattern, to promote the city cluster as a whole coordinated and rapid development.

Third, transportation integration should be used as a chance to lower the overall city cluster distance cost. To prevent distance expenses to a greater extent and limit the development of the city group inside the Guangdong-Hong Kong-Macao Greater Bay Area, we should focus on the improvement of transport infrastructure to improve the overall efficiency of the internal transport network. Since 2018, the development of the Hong Kong leg of the Guangzhou-Shenzhen-Hong Kong Express Rail Link, the Hong Kong-Zhuhai-Macao Bridge, etc., has substantially decreased the travel time with Hong Kong and Macao, "one-hour living circle" is just around the corner. The "one-hour living circle" is just around the corner. Regional cities on the edge need to take this development opportunity to facilitate exchanges and cooperation with core cities, and at the same time actively reduce the spatial transaction costs between edge cities and core cities, improve the overall access efficiency of the Guangdong-Hong Kong-Macao Bay Area, reduce the distance cost hindrance to a minimum, and realize long-distance cooperation, to promote the core cities to better play the trickle-down effect, enhance the overall level of the Guangdong-Hong Kong-Macao Bay Area, and better anchor in the globalization of digital information. In the wave of the development of the general trend of global digitalization.

### Research limitations

This paper adopts five factors in constructing the regression model of information network influencing factors. It achieves a better regression effect, but the actual influencing mechanism within the urban agglomeration is more complicated. This paper is based on a measurable and quantifiable standard. When screening some influencing factors that may tend to influence, some of the influencing factors have been discarded because of the difficulty of finding them or the lack of a quantitative mechanism, so the regression model of this paper is still lacking and not comprehensive enough. The analysis of the information network’s influence mechanism and driving force still needs further attention from scholars to be enriched.

## Supporting information

S1 Fig Visualization structure of the change of information linkage intensity in the Guangdong-Hong Kong-Macao Greater Bay Area city cluster, 2012–2021. Contains all data for Fig 1.
(DOCX)

S1 File (DOCX)

S1 Table Contains all data (the Information Network Matrix, data sets of factors and 5 variable influencing information linkages) for Tables 1–6. (DOCX)
