# Supplementary material for: Evolution characteristics and influencing factors of information network in Guangdong-Hong Kong-Macao Greater Bay Area
Source: PLoS One. 2024 May 17;19(5):e0298410. doi: 10.1371/journal.pone.0298410 (PMC11101075; doi:10.1371/journal.pone.0298410)

**Supplemental Material**

**Figure 1 Data Set:** information linkage intensity in the Guangdong-Hong Kong-Macao Greater Bay Area city cluster in in 2012, 2015, 2018 and 2021.
**2012**


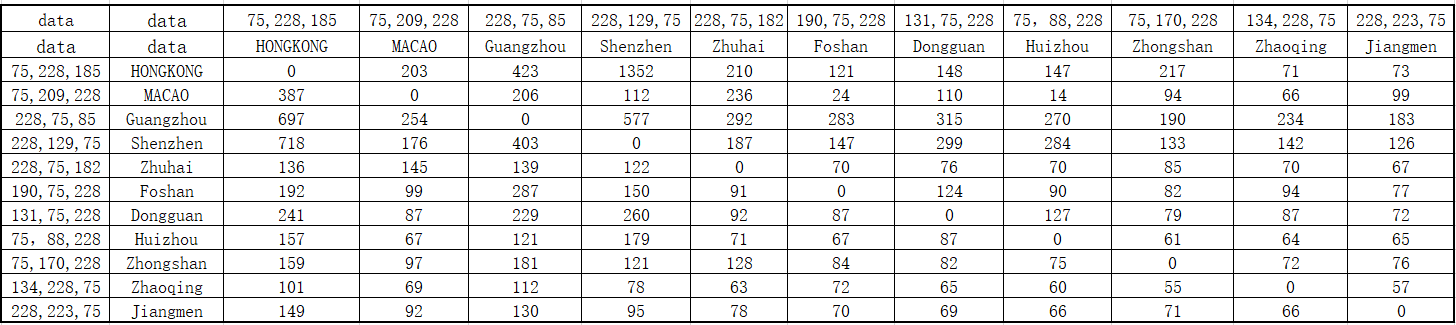


**2015**

**
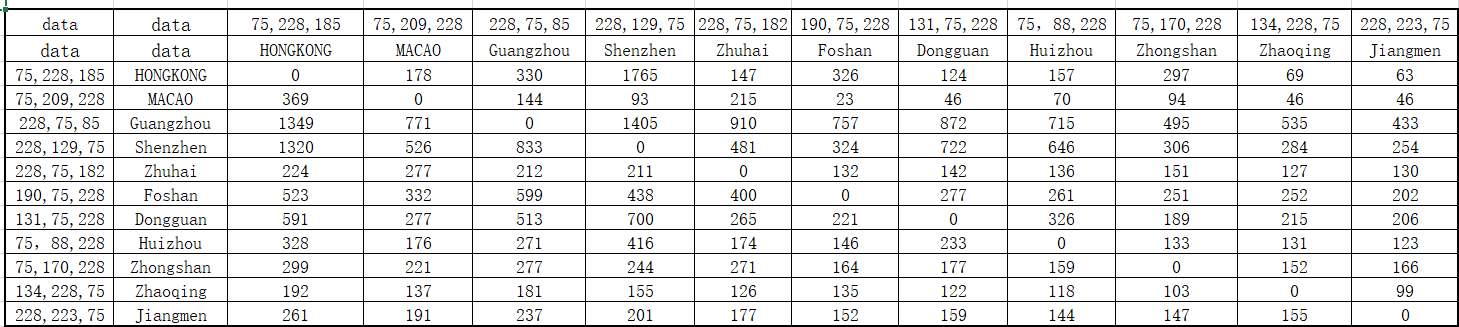
**

**2018**


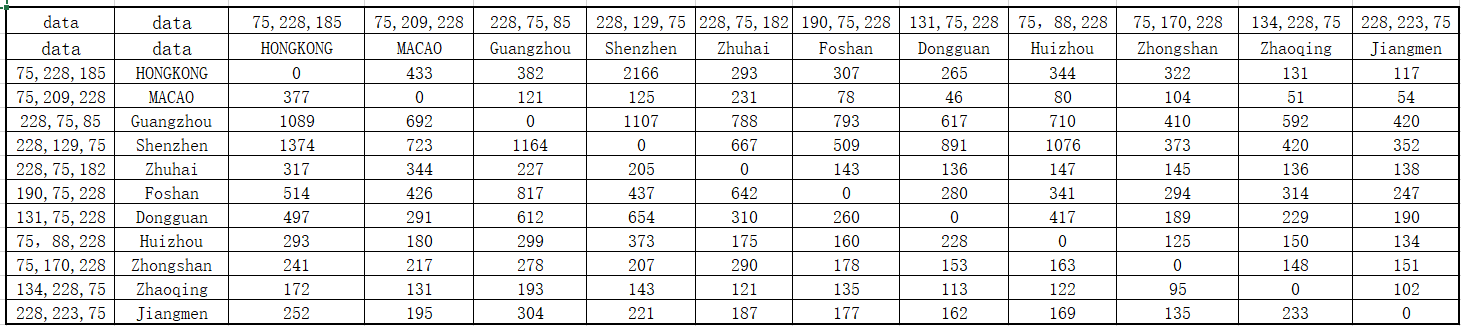


**2021**


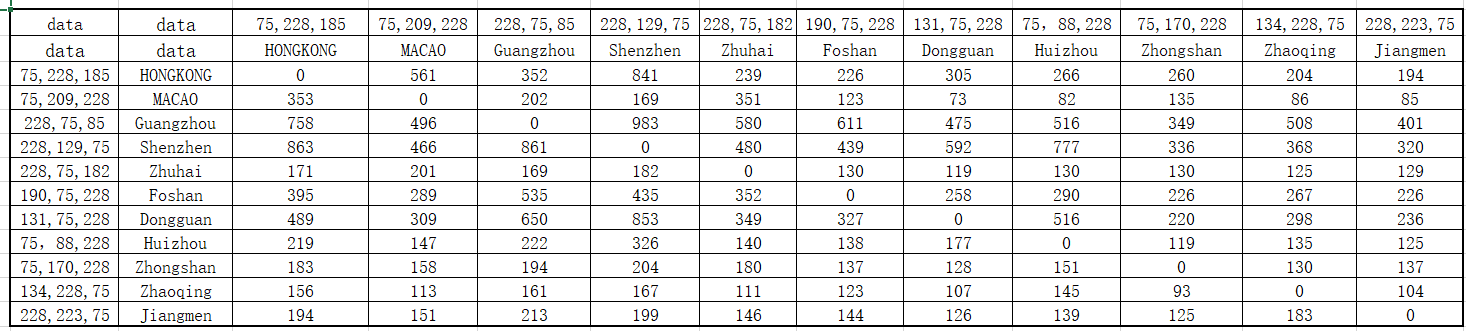

Supplement: S1 Fig — Contains all data for Fig 1. (DOCX) [file pone.0298410.s001.docx]
