# Supplementary material for: Evolution characteristics and influencing factors of information network in Guangdong-Hong Kong-Macao Greater Bay Area
Source: PLoS One. 2024 May 17;19(5):e0298410. doi: 10.1371/journal.pone.0298410 (PMC11101075; doi:10.1371/journal.pone.0298410)
Supplement: S1 File — (DOCX) [file pone.0298410.s002.docx]

For obtaining Baidu Index data and Google Trends data for each city in Guangdong, Hong Kong, and Macao Greater Bay Area, we used the relevant functions of the Baidu Index (https://index.baidu.com/v2/index.html#/) and Google Index website (<https://trends.google.com/trends/>). Specifically, we search on the Baidu Index and Google Index websites by typing the name of a city, such as Guangzhou, into the search box and then pressing the Enter key. After entering the new page, we click the "Compare" button after the keywords to add other cities and adjust the data period to 2012-2021, to obtain the Baidu Index and Google Trends data of each city in Guangdong, Hong Kong, and Macao Greater Bay Area.

Go to the official website of the China National Bureau of Statistics (<http://www.stats.gov.cn/>) and type "China Statistical Yearbook" in the search box at the top right corner to get the relevant data from 2012-2021. Specifically, in the annual China National Statistical Yearbook, we can find data on the number of employed people and the number of cell phone subscribers in Hong Kong and Macau, e.g., after clicking on the 2021 China Statistical Yearbook, data on Hong Kong can be accessed in section 26, and data on Macau can be accessed in section 27.

Visit the website of the Statistical Yearbook Column for Guangdong Province and 21 cities (<https://gdzd.stats.gov.cn/dcsj/gdsnjsj/201902/t20190201_154503.html>). We can obtain data on the number of employed population, urbanization rate, number of cell phone subscribers, R&D research expenditure, etc. for the other 9 cities in the Guangdong-Hong Kong-Macao Greater Bay Area (Guangzhou, Shenzhen, Zhuhai, Foshan, Dongguan, Huizhou, Zhongshan, Zhaoqing and Jiangmen), except Hong Kong and Macao. Taking Guangzhou City as an example, we could click on Guangzhou Statistical Yearbook (2000-2023) to access the official website of the Guangzhou Municipal Bureau of Statistics where the 2012-2021 Statistical Yearbook is stored. On that website, we can access data on the number of employed people, urbanization rate, number of cell phone subscribers, R&D and scientific research expenditure in Guangzhou from 2012 to 2021.

Visit the official website of the Census and Statistics Department (<https://www.censtatd.gov.hk/sc/>), click the "Statistics" button on the upper left to enter a new page, and then click "Web Tables". "And then click Table 710-86001 in the web tables to find the R&D funding data of Hong Kong from 2012 to 2021.

Finally, visit the World Bank public data (<https://data.worldbank.org.cn/>) and the official website of the Statistics and Census Service of Macao (<https://www.dsec.gov.mo/zh-MO/>). Through these two websites, we can obtain data on the urbanization rates of Hong Kong and Macao from 2012 to 2021, as well as data on the number of employed people in Macao.
