# Supplementary material for: Evolution characteristics and influencing factors of information network in Guangdong-Hong Kong-Macao Greater Bay Area
Source: PLoS One. 2024 May 17;19(5):e0298410. doi: 10.1371/journal.pone.0298410 (PMC11101075; doi:10.1371/journal.pone.0298410)

**Supplemental Material**

**Table 1,2,3 Data Set:** Study of Centrality Analysis and Eigenvector Analysis and Core-Edge Analysis of the Information Network Matrix of Guangdong, Hong Kong, Macao and the Greater Bay Area in 2012, 2015, 2018 and 2021

**2012**


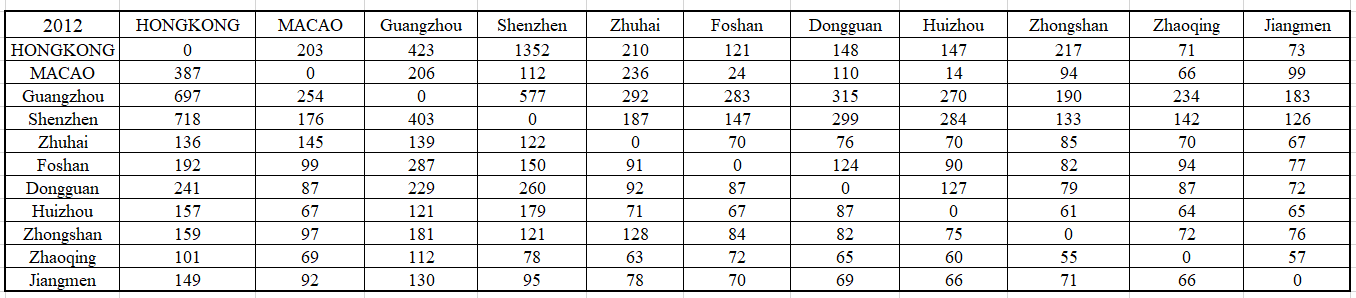


**2015**


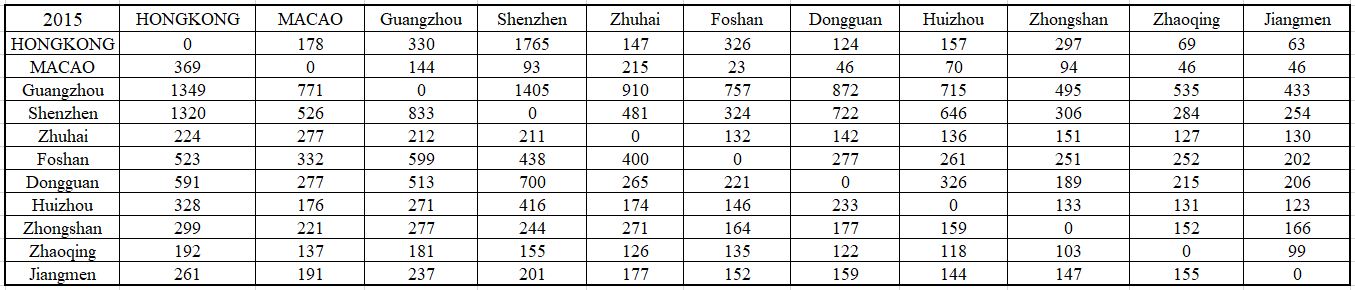


**2018**


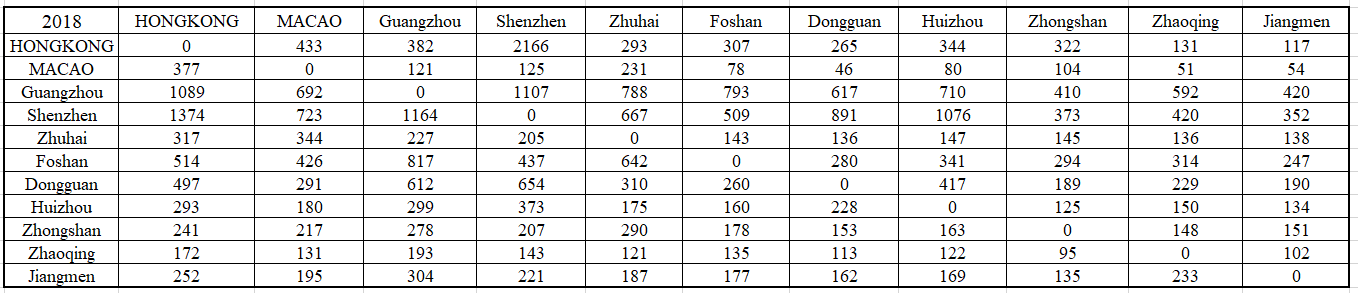


**2021**


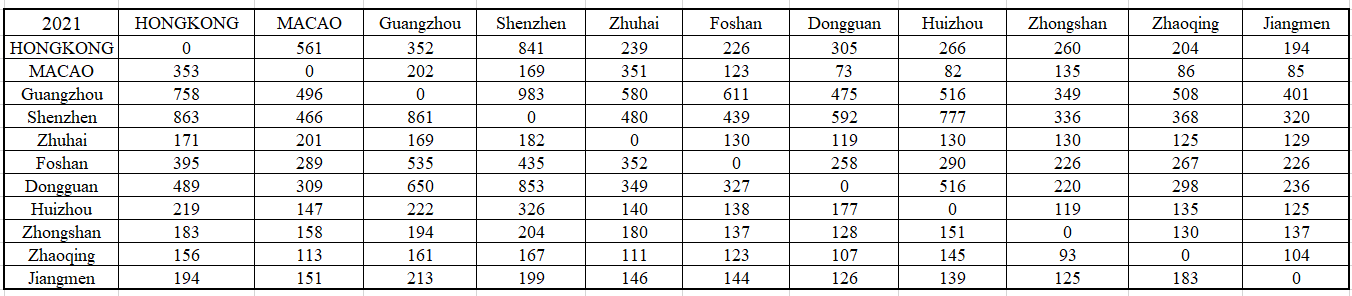


**Table 4 Data Set:** data sets of factors influencing information linkages in the Guangdong-Hong Kong-Macao Greater Bay Area city cluster


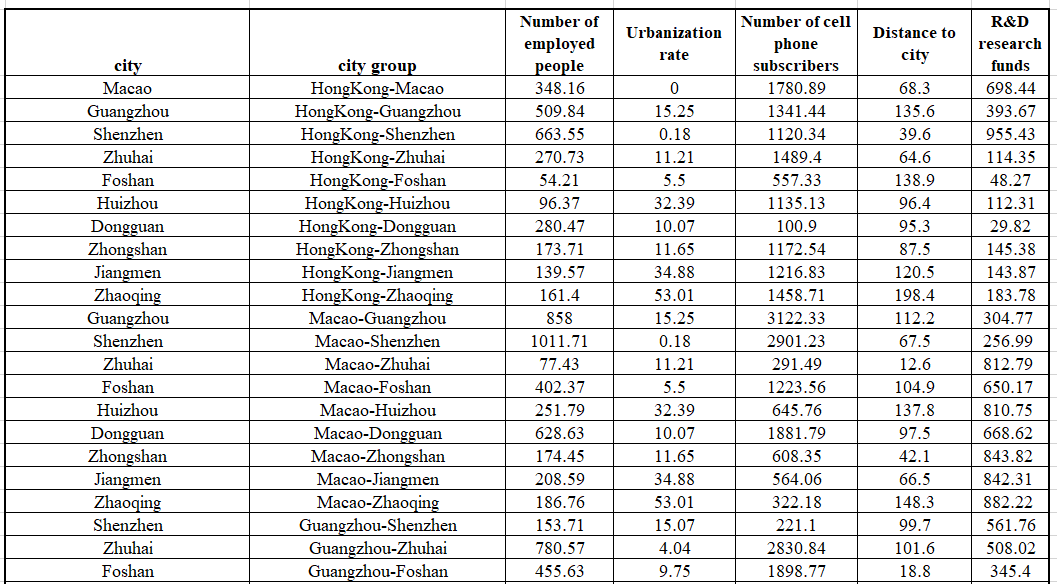


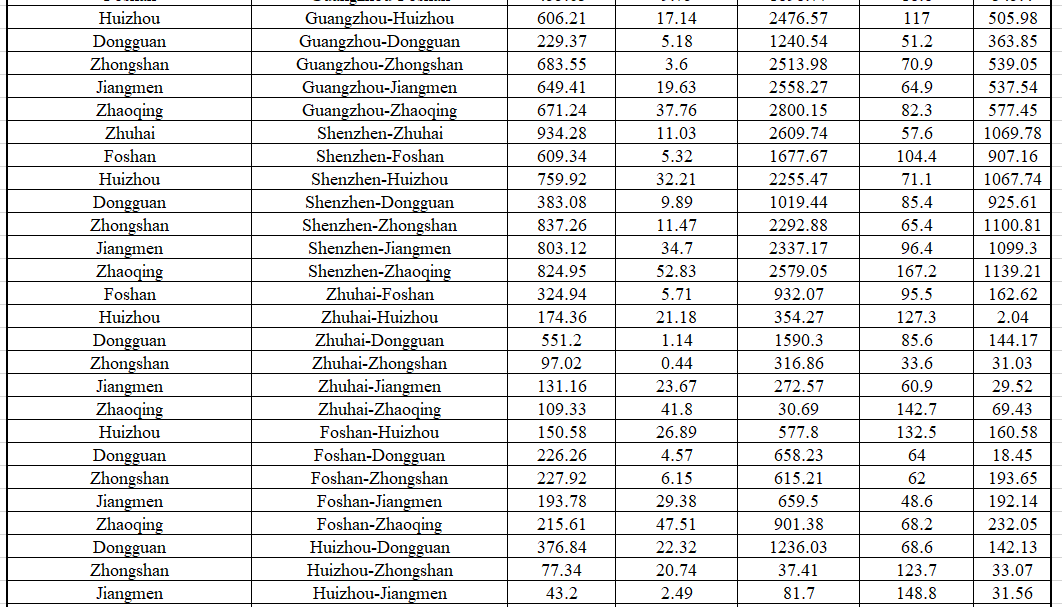

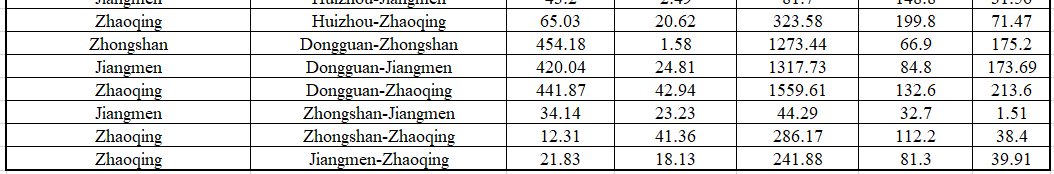


**Table 5,6 Data Set:** Data sets for each variable of QAP regression and correlation analysis of the factors influencing the information linkage network of Guangdong, Hong Kong and Macao Greater Bay Area

**Number of employed people**


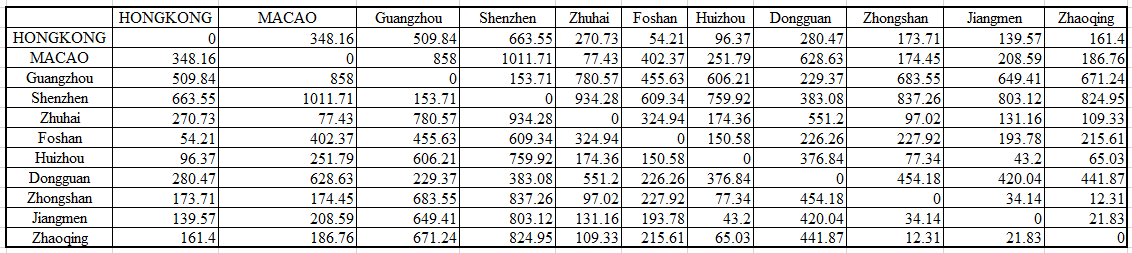


**Urbanization rate**


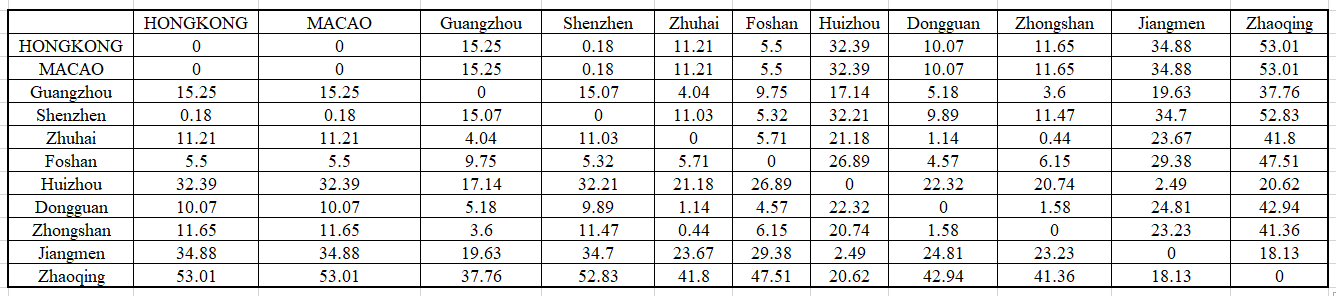


**Number of cell phone subscribers**


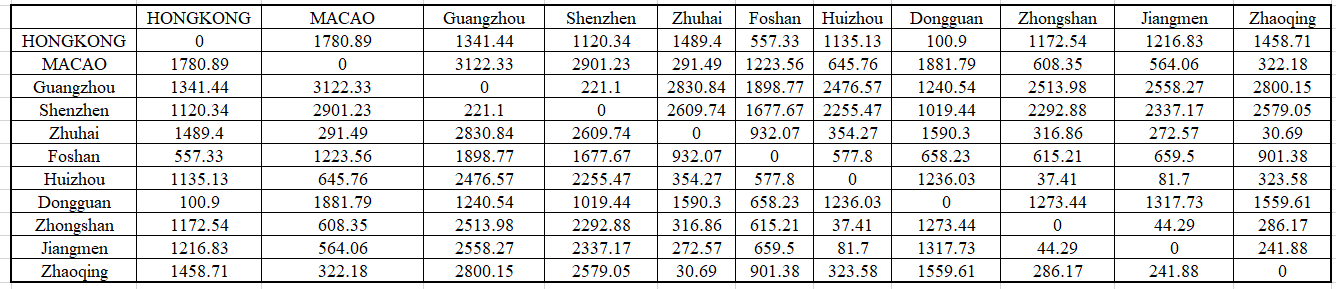
 **Distance to city**

**
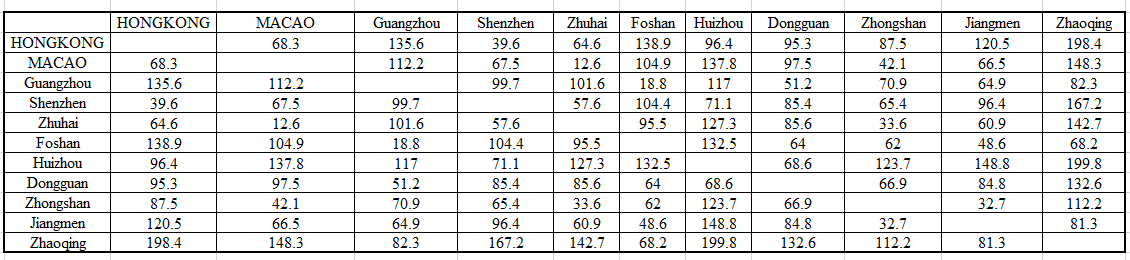
**

**R&D research funds**


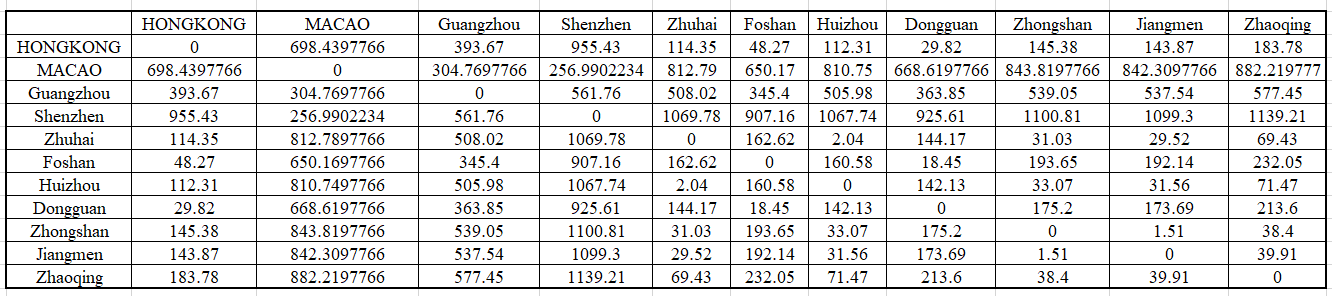

Supplement: S1 Table — (DOCX) [file pone.0298410.s003.docx]
